# Using collaborative autoethnography to explore the teaching of qualitative research methods in medicine

**DOI:** 10.1007/s10459-023-10224-z

**Published:** 2023-04-27

**Authors:** Kinda Ibrahim, Susie Weller, Elissa Elvidge, Meredith Tavener

**Affiliations:** 1 School of Primary Care, Population Science, and Medical Education, Faculty of Medicine, University of Southampton, Southampton, UK; 2 National Institute of Health and Care Research (NIHR) Applied Research Collaboration (ARC) Wessex, Southampton, UK; 3 Clinical Ethics, Law and Society - Southampton, Faculty of Medicine, University of Southampton, Southampton, UK; 4 Clinical Ethics, Law and Society - Oxford, Wellcome Centre for Human Genetics, University of Oxford, Oxford, UK; 5 School of Medicine and Public Health, College of Health, Medicine and Wellbeing, University of Newcastle, Newcastle, NSW Australia

**Keywords:** Collaborative autoethnography, Medicine, Pedagogy, Qualitative research methods, Teaching and learning

## Abstract

This article explores experiences of teaching qualitative research (QR) broadly, and qualitative methods (QM) more specifically in medicine, highlighting the challenges faced, and offering recommendations for overcoming them. Using collective online interviews, collaborative autoethnography (CAE) was employed to generate data comprising educator’s reflective accounts of teaching QM in medical schools across two continents. Three main themes were identified through collaborative thematic analysis: making meaningful contributions from a marginalized position; finding our pedagogical feet; and recognizing the translational applicability and value of QR. We reflected on the marginalized positioning of QM in medical curricula and the underestimation of the value of QR to understanding pressing health issues. Analysis of these reflections pointed to a lack of formal training for educators and curriculum space for qualitative approaches. Our teaching pedagogies, developed through our own research experiences, self-reflection and student feedback, were primarily student-centered employing a range of novel approaches designed to foster skills and interest in the craft of QR, and introduce a greater appreciation of the significance of QR approaches to medicine. CAE further allowed us to identify some key recommendations that could help medical educators plan for teaching QM and other research methods more effectively in medicine. Future curriculum development should consider the benefits of exposing learners to a range of methods and approaches from across the qualitative-quantitative spectrum.

**Supplementary Information:**

The online version contains supplementary material available at 10.1007/s10459-023-10224-z.

## Introduction

Qualitative research (QR) is concerned with “the way people interpret and make sense of their experiences and the world in which they live” (Jindal et al., 2015). QR has increasingly been acknowledged as integral to understanding the most pressing health concerns and, therefore, important in medical education (Rolfe et al., 2018; Renjith et al., 2021). For example, QR provides a means of examining the social, emotional, and psychological aspects of care, with research outcomes shaping clinical practice. However, incorporating QR approaches in a meaningful manner into medical curricula continues to prove challenging (Eakin & Mykhalovskiy, 2005; Sawatsky et al., 2019). In both under- and post-graduate research methods courses there is often limited coverage of qualitative methodologies and methods (QM) (Eakin & Mykhalovskiy, 2005). Where there is a focus on QM, learning opportunities in higher education are often limited to short, compulsory classes that provide a cursory overview of a limited number of approaches, with little time for in-depth engagement (Katz, 2015). This is especially problematic in Medicine given that many medical students are unlikely to be familiar with QR and QM, with much of their previous/current training is dominated by positivist and post-positivist approaches and quantitative methods of inquiry (Breuer & Schreier, 2007). Given that QR can shed light on a range of issues of pertinence to contemporary medicine, students would benefit from gaining a greater sense of the breadth, application and value of QR to both research and practice.

There is also a lack of pedagogical support for QM with many educators in medicine having not received formal training (Eakin & Mykhalovskiy, 2005; Delyser, 2008). Rather their practice is shaped by their own experiences of conducting QR, relying on the literature, peers and/or experimentation, which Nind and Lewthwaite argue stems from a paucity of pedagogic research more generally (Nind & Lewthwaite, 2018). QR comprises a range of epistemological perspectives, with researchers drawing on a variety of conceptual frameworks and employing different methods and tools. This range is often not reflected in what is delivered to medical students due to educators facing curricula and time constraints on one hand and the lack of educator training and diversity in teaching approaches and tactics on the other hand (Poulin, 2007; Roulston & Bhattacharya, 2018). Work done by Eisenhart and Jurow showed the different approaches taken by educators, with some focusing on research design and the application of different methods, while others prioritize epistemological issues (Eisenhart 2011).

This article presents a synthesis of stories of learning and connection from our experiences of teaching QM in two universities in Australia and the UK. In bringing together these examples, it is essential to reflect on the salience of the broader context in which we are working, particularly, in medicine. Our experiences are shaped by an increasingly market-oriented environment, which has seen a shift to positioning knowledge as a ‘commercializable commodity’ (Weller, 2022; Lyle et al., 2022) and students as consumers. An idealized student ‘experience’ is regarded as the desired outcome and necessary for the generation of further revenue, with teaching quality, including QM courses, assessed using market-driven metrics (Cannella & Koro-Ljungberg, 2017). Whilst such reforms have re-framed relationships between institutions, educators, researchers and learners, there are also implications for the ways in which different kinds of research are valued. For instance, Edwards (2022) talks of researchers’ concerns about “the prioritizing of quantitative over qualitative methods in a neo-liberalized climate”. It is against this shared backdrop that we explore our experiences of teaching QM.

This article focuses on the outcomes of a collaborative autoethnographic (CAE) endeavor, the aim of which was to reflect critically on our experience as educators teaching QM in medical schools focusing on the challenges faced and the steps necessary to overcome them. CAE is a multivocal approach whereby two or more researchers share and pool stories, and explore, unpack, and synthesize individual and collective experiences (Chang et al., 2013, Blalock & Akehi, 2018). Stemming from autoethnography (Ellis, 1991; Ellis & Bochner, 1996), CAE data comprises self-narratives and reflections that can be generated through a variety of means including, as used in this article, collective interviews (Roy & Uekusa, 2020). It is an iterative, systematic process comprising ongoing discussions, critical reflection and analysis of personal experiences with meaning-making situated at the individual and collective levels (Denzin, 2014). The key aim is to foster ‘intentional and purposeful dialogue’ (Blalock & Akehi, 2018: 90). Interest in the use of approaches employing autoethnography grew during the COVID-19 pandemic in response to infection control measures that restricted more conventional in-person QR (Roy & Uekusa, 2020). Yet, the experiences of educators have long been useful in shaping practice. For example, in reflecting on psychology, Walsh-Bowers (2002) argues that “One useful source of evidence on the present and future status of qualitative methods in psychology is investigators’ accounts of their own experience as researchers, authors, teachers, supervisors, and students” (Walsh-Bowers, 2002). For us, CAE provided the means to confront, share and make sense of our experiences of teaching QM in medicine. Therefore, the purpose of this article is to illuminate the challenges educators often face, as well as the successes so that both students and educators might benefit from a broader and more in-depth understanding of value of QR and QM to research and practice.

## Research context and design

In response to changing research practice during the COVID-19 pandemic, an international workshop on the teaching of QR online was convened by authors, Kinda Ibrahim [KI] and Meredith Tavener [MT], in October 2020. This idea for this article stemmed from workshop discussions concerning the need for critical dialogue about QM teaching practice and the desire to forge opportunities to explore feelings, uncertainties, and creativities regarding the teaching of QM to students likely to have different ontological assumptions and, perhaps little interest in QR. Both KI and MT are experienced in conducting QR and in designing and delivering postgraduate QM courses for medical students and clinicians, many of whom are unfamiliar with such approaches, in the UK and Australia. Authors EE and SW, both social scientists working in health sciences and medicine in Australia and the UK respectively, were invited to join the collaboration.

In the spirit of CAE, the team worked iteratively and systematically, holding ongoing discussions and critical reflections over time. CAE was deemed appropriate as we sought to share, compare, and understand our own experiences of teaching QM in different contexts. In developing our CAE approach, we spent time exchanging reflections on how our own positionalities shaped the kind of QR we conduct, how we teach QM, and the assumptions we make about the learners with whom we engage (see Box 1).

Box 1. Our positionality Kinda Ibrahim (KI) is an applied health researcher with undergraduate training in pharmacy which used dominantly quantitative and lab-based approaches. KI has over 11 years’ experience in teaching and using QR. In 2018, she established an international qualitative network to facilitate a peer support environment to help build the knowledge and capacity of researchers in QM. KI teaches QM as part of research methods modules to both undergraduate medical students and postgraduate candidates studying various healthcare professional degrees. Qualitative teaching in this course is a part of wider education about different research methods including randomized controlled trials, cohort study design, and systematic reviews. KI’s pedagogical approach is underpinned by discipline-relevant pedagogy and the realist/pragmatic paradigm that she employs in her own applied health research. KI’s epistemological perspective is informed by and combines both interpretivism and pragmatism. Her teaching is driven by the desire to provide students with the knowledge and skills that enable them to use QR in their clinical practice. On reflection, her teaching is influenced by the time allocated to QR in her course, the characteristics of her students coming from fields dominated by positivist and post-positivist approaches, her own research practices as an applied health researcher, and by her previous experience as an undergraduate student taught in a quantitative-dominant environment.

Meredith Tavener (MT) gained degrees in exercise physiology, health promotion, clinical epidemiology and social gerontology, and has 20 + years in health research, 12 of which have involved teaching QM (in Australia and the Netherlands). Despite receiving high distinctions when studying Biostatistics MT never felt confident with numbers, and gained deeper satisfaction from the human exchange inherent to much QR. Since 2019, MT has coordinated and delivered her QM in health research course, taught online to post-graduate students, biannually. Her curriculum and teaching approaches embrace her constructivist approach, focusing on collaborative empowerment and student competencies via research-led learning MT offers different delivery pathways and assessment types and provides practical qualitative experiences to acknowledge different student realities of learning. As a cis female who conducts research with not-for-profit and/or disability advocacy organizations, MT is conscious of the ‘voices’ she can involve and bring forward through her own research. Her course is structured towards promoting conceptual development in students and increasing student confidence in QM.

Susie Weller (SW) is a senior researcher with over 20 years’ experience of QR and teaching QM. From a working-class background, she was the first of her family to attend University. SW is passionate about promoting the value of qualitative approaches to understanding social processes. SW’s undergraduate degree was in Geography through which she was exposed to the broad spectrum of qualitative and quantitative methods and approaches. Over time, she has specialized in QR and has long-standing interests in qualitative longitudinal, creative, and participatory approaches, qualitative secondary analysis, and ethical research practice. Framed by feminist social constructionism, much of her work has been in interdisciplinary teams straddling the social and biomedical sciences. Researching and teaching in interdisciplinary teams has provided SW with many opportunities to question her own assumptions about what constitutes qualitative and quantitative work. This has been most evident in her recent development, with colleagues, of a new method for big qual analysis that combines computational techniques with more conventional forms of qualitative analysis (Davidson et al., 2019) SW has published over 25 methods-oriented papers. She has taught QR and QM at both under- and post-graduate levels along with introductory courses for biomedical scientists and advanced courses for experienced researchers.

Elissa Elvidge (EE) is a post-doctoral research fellow with the University of Newcastle School of Medicine and Public Health. EE’s work is primarily grounded in critical race theory, feminist and decolonizing theoretical perspectives. Her research focuses on cultural safety in the health system and in higher education. Elvidge is passionate about QR and highlighting individual stories to find those common threads that make up shared experiences across institutions. Although EE has taught sociology and across multiple health disciplines, she has not had any experience of specifically teaching QR. This insider outsider perspective gives her the ability to move between an objective/ subjective point of view and analyze the interviews from a position of awareness of her positionality.

### Data generation

Data generation comprised a series of in-depth interviews along with more informal group discussions and reflections. At the outset, KI and MT wanted to find space in which to explore their own feelings, uncertainties, and creativities regarding their own pedagogical influences and experiences of teaching QM to medical students. KI’s undergraduate background was heavily dominated by training in quantitative approaches, however her interest in patient’s experiences prompted her to pursue postgraduate training in QR and applied health research. MT’s diverse health background brought with it a large portion of training in quantitative approaches, though MT always sought to focus solely on QR. To formalize conversations, a third colleague, SW, with significant experience in QM was invited to facilitate the CAE by designing and conducting the interviews. SW brought her expertise as a social scientist to the endeavor. SW’s work has encouraged her to be cognizant of the way dominant epistemologies in different (sub)disciplines shape how QR is construed, undertaken, and valued. Our fourth colleague, EE, was introduced to the group by MT, and tasked with analyzing the interviews. EE brought her own experiences and reflections of being a woman of color and early career researcher working in an Australian health service and university context. Although originally intended to capture exchanges between KI and MT, the CAE evolved with reflections from EE and SW increasingly shaping the discussions. The first three, more formal interviews took place online between January and March 2021, attended by three authors (KI, MT and SW) with author (EE) joining from the second interview onwards. Each was video recorded via Microsoft Teams and the live transcript saved and checked.

Semi-structured interview guides were used (see supplementary file for interview 1 topic guide), which galvanized with each interview. The interview time for the three primary discussions totaled 4 h, 14 min and 51 s (1.57.23, 1.20.16 and 57.12 for interviews 1, 2 and 3 respectively). The content of the topic guides was informed, in part, by the conceptual-empirical typology of social science research methods (Nind & Lewthwaite, 2018, 2020). Although designed for, and with those teaching advanced research methods in the social sciences, it has wider application as a tool for thinking about and developing pedagogic practice. The typology focuses on four aspects of pedagogy: (i) the philosophies or values underpinning the educator’s *approach;* (ii) the *strategies* adopted to deliver the approach; (iii) the *tactics* employed to convert the strategies into practice and respond to factors arising in context; and (iv) the *tasks* or activities assigned to learners. Accordingly, the interviews sought to understand pedagogical practice. We focused on our own journeys through learning QM, our teaching histories, experiences, and pedagogical approaches; how we conceived of our learners’ diverse interests and needs. We also discussed the value and place of QR within the disciplines, departments, and faculties in which we are located including reflections on perceptions of dominant epistemologies, methodologies, and pedagogies, along with the influence of the wider context shaping our experiences of HE. We presented our approaches to designing courses, sessions, and modes of assessment; perspectives on ‘what works’ in practice as well as constraints. We reflected on the inter-connections between teaching and research; the training needs of educators and our colleagues; and future imaginings of QM pedagogy in medicine.

### Data analysis

The interview transcripts were analyzed using a collaborative ethnobiographic reflective lens in an iterative process of critical-reflection, discussion, and collaboration (Méndez, 2013). All contributed to the process, each bringing different subjectivities, interests, and disciplinary backgrounds to the analytic endeavor. We also kept an online journal to capture individual or shared reflections, thoughts, and ideas we had during the process. These reflections were used as a source of data alongside the interview transcripts and our more informal discussions. The data were analyzed thematically, working recursively initially across three cycles each representing the more formal interviews. In conducting her analysis, EE was guided by a reflexive approach to thematic analysis (Braun & Clarke, 2019. Both semantic and latent coding occurred with EE labelling chunks of text with similar meanings. A list of main codes was then presented to KI, MT, and SW in a virtual meeting to discuss the findings and reflect on whether they represented their experiences and stories. A fourth cycle of analysis accounted for the online journal notations and four lengthy informal discussions. These discussions were recorded and used in the analysis to explore additional insights and deeper reflections. We acknowledge that in discussing and refining codes and themes, we brought back with each iteration, our own viewpoints and what we held as our own erudition within QR. In the reflexive analysis, emphasis was also placed on multiple and/or contradictory narratives within each account. Themes were constructed from patterns of shared meaning (Braun & Clarke, 2019). The whole team was involved in the analytic process including deciding on the plot of the collective story, any contradictions, the development of themes and the selection of illustrative quotes. Planning, regular communication, respectful relationships among team members, and methodological innovation were critical to ensuring the quality of this work.

## Findings

Drawing on 12 h of discussion and reflection generated over one year, we discuss three main themes identified and constructed through our analysis: (i) making meaningful contributions from marginalized positions; (ii) finding our pedagogical feet; and (iii) recognizing the translational applicability and value of QR.

### Making meaningful contributions from marginalized positions

Our CAE work reiterated the feeling that QR inhabits a marginalized position in medical education. This, in part, stems from how we believe that others perceive QM in terms of their potential to make meaningful contributions to the kind(s) of knowledge valued in such disciplines, as well as understandings of scientific rigour. Our own discussions were peppered with examples of the challenges (and sometimes, exasperations) faced, that we often attributed to enduring tensions between our work environments where positivist and post-positivist methodologies dominate under- and postgraduate programmes and our efforts to illuminate the value of interpretivist approaches and those drawing on critical theory (Bunniss & Kelly, 2010). KI described such challenges:

‘We try and make them switch their mindset to a different method, a different mindset and way of thinking …’ (KI).

Along similar lines, MT asks her students to reflect upon their positioning and assumptions, and then discussed “… *retraining your brain…*” towards a more qualitative mindset. For QM educators, this is especially challenging as not only are we required to teach a range of complex interpretive and critical concepts and skills, but there is also the added hurdle of encouraging students to acknowledge and challenge their own preconceptions of QR as, less scientific, or even less useful to their current studies and/or future professions, than the approaches and methods with which they are more likely to be familiar.

In describing the challenges of teaching QM, KI and MT referred to an enduring hierarchy of research methods, that they themselves had been introduced to many years prior, in which quantitative approaches were favoured and positioned as higher status or more ‘credible’ whilst QM were undervalued and perhaps misrepresented. When referring to such a hierarchy, SW commented that ‘*…its already set up that way, what is valid and valuable’*. In so doing, she drew on Edwards & Holland (2020, p. 906) aforementioned point regarding researchers’ concerns how, in neo-liberal contexts, quantitative methods are afforded greater precedence.

Teaching QM to medical students often felt like an undervalued pursuit. Within an HE context where accountability and surveillance increasingly feature, we shared our constant struggle to prove the legitimacy of such courses to our institutions, faculties, colleagues, and students. This conflict was characterised by KI and MT as an uphill battle where their individual expertise was often un(der)appreciated by colleagues and the significance of such work marginalized. Throughout our discussions, both KI and MT characterised a constant friction omnipresent during their teaching, using somewhat combatant language such as ‘defending’, ‘punch’ and ‘fighting’, to signify the prolonged campaign in which they have engaged to gain recognition of the legitimacy of interpretivist approaches and those employing critical theory and their place within their courses:

‘You are in a position where you are always defending the methods’ (KI).

‘… it takes us so much work… to wave and cheer … and say do you know what we do for a living is actually valid, it’s incredibly important… It’s incredibly technical and complex. It doesn’t have to be one way there can be another way – we are just fighting for it all of the time’ (MT).

Connected to the ‘usefulness’ ascribed to QR, KI and MT shared stories of feeling their labour was undervalued. Both were amongst just a few people within their departments able support students and colleagues with QR and they were regularly asked to undertake additional, unrecognized, and unpaid work. The people asking for such advice seemed satisfied with the exchange, but MT spoke of how long it could take, and that consequently other work was pushed aside:

‘Once people know what you can do and how easy you are to speak to…you can see them physically relax and go oh, I didn’t know that, I’m so glad that I spoke to you. But of course, they’ve spoken to you for at least an hour and then they’ve gone’ (MT).

MT’s example highlights more hidden forms of teaching, such as informal mentoring, performed often by QR experts in departments and faculties where such work is often marginalized. Our passion for QR was a key motivator driving our willingness to teach and support others, particularly our desire to ensure that the value of QR is recognized in our departments and faculties and more widely:

‘In terms of the teaching, the driver is really my passion about educating people and making people aware of the methods and to make them think that this is a valid method to teach in or to use in research and especially in healthcare research’ (KI).

The casualization of employment in HE in both the UK and Australia means that such hidden forms of labour may be completed at the expense of other career-enhancing tasks and are likely to be particularly detrimental to the increasing numbers of academics on short- or part-time employment contracts.

### Finding our pedagogical feet

Our first discussion commenced with recollections of the serendipitous circumstances that led us to teaching QM. MT was ‘head-hunted’ specifically to join an existing team of public health educators in her School, to teach QM in health research. MT started with… “*the notes from the previous person, and I just rewrote it not knowing at all what I was doing…. I started to teach.’* (MT). KI nodded and followed with a similar story of learning on the job: ‘*It’s the same with me. No one told me or taught me what to do or how to teach QR.’* (KI).

Both had completed under- and post-graduate degrees in health-related disciplines and had not undertaken formal training in teaching QM. Both described a similar process of being self-taught, with the focus of their teaching scaffolded by their own experiences as qualitative researchers. As the conversation progressed, SW asked how they viewed themselves as educators, with both hesitating before answering. The use of CAE helped the authors reflect on and draw out salient experiences and practices they had not previously considered:

‘When I started, I had no idea what teaching was, so I brought all of my research bad habits with me and just thought I’m just going to share them and be enthusiastic… So in my committee meetings with my colleagues, they would talk about certain pedagogical frameworks and philosophies and authors and I’m sitting there writing down words thinking what the bloody hell is that? I had to reflect on why I did things a certain way and who I was trying to be…‘. (MT)

KI went on to describe her approach to teaching:

‘I teach QR the way I do research. So, I get them into a journey of how to do QR. These are the kind of questions that you can answer. This is how we choose our sample. This is how many people we need in our sample. This is how we do our interviews, so I give them the skills of doing the interviews or the focus groups or the observation. This is how we do the analysis … This is how we present the data. So, it’s like teaching someone who’s never had the experience before in QR, how to do a simple piece of qualitative work‘. (KI).

Both MT and KI described how they developed their teaching pedagogies, based on self-reflection and student feedback as they taught their courses. MT characterised her approach as focused on real-world problems and scenarios, student-centered, reflective and critically reflexive, having to change between critical or ‘avant-garde’ when the situation demanded. She emphasized epistemological and ontological principles from the first week of teaching. Finding that her postgraduate students were unaccustomed to introspection, time was invested early in MT’s course on strategies to encourage students to consider ‘how they see the world’ and using visual inquiry tasks such as “I see… I think… I wonder… “. MT teaches her course biannually, which provides up to 13 weeks for deeper dives into advanced qualitative concepts and epistemological soundness. Conversely, KI saw her approach as pragmatic. Governed largely by the limited time afforded to QM in her student’s courses (one week for the taught postgraduate course and one day for undergraduate courses), she spoke of taking a prescriptive and pragmatic strategy to instilling a limited range of methodologies, methods and skills that could then be applied to a simple QR project and/or clinical practice. Teaching pharmacists, doctors, allied health professionals, her pedagogical approach is active, experiential, and student-centered and underpinned by the discipline-relevant pedagogy and realist/pragmatic paradigm that she employs in her own applied health research.

In terms of teaching online, these different teaching styles and QR pedagogies needed to be reconciled against changing student expectations. KI and MT discussed online post-graduate course design, and how it may enable greater numbers of clinicians and health professionals to study QM. KI had to shift to teaching online during the pandemic. Although this allowed greater time to be allocated to QM, she found virtual teaching was inflexible and challenging. KI had to adapt in-person tasks and align them with her pragmatic teaching style. For example, she replaced lectures with pre-recorded sessions that comprised integrated quizzes and activities, live sessions with break-out rooms for group work on designing topic guides and coding exercises and used online fora and drop-in sessions for answering questions. Conversely, MT’s QM teaching had always been delivered online, and she embraced the flexibility in expression that this afforded (e.g. coding via iPad onscreen and micro-lectures) plus the ability to have more one-on-one student interactions. With MT’s learners being employed or upcoming health professionals she focused specifically on introducing opportunities for authentic learning or applied work (e.g. ethics or grant applications).

Even though both KI and MT had slightly different approaches to teaching, there were many commonalities, that became apparent during the CAE, especially their shared student-centered approach.

KI: “I really like the idea that QR is taught by active engagement with the students.”

MT: (nodding in agreement with KI) “Yeah, I find that my students are most fascinated by coding. It’s just up to them (but they) sometimes have no idea what on earth they’re supposed to do. So, a lot of (my coding) are micro lecture examples that are pre-recorded.

KI: “The way we teach QR for healthcare professionals who are working in practice is different from the teaching I do for medical students…. I always try to use examples that fit in with their interests.”

KI and MT described their focus on experiential learning, emphasizing the different techniques they employed to help foster a greater appreciation of the complexity and value of QM and the relevance to healthcare contexts and clinical practice. Both spoke of offering opportunities for students to develop skills that would serve the dual purpose of conducting QR but also could be applied in a broader health context. Tasks included an exercise to identify one’s conceptual underpinnings, role play activities to practice listening and interviewing skills, coding exercises using existing data, visual inquiry, or analysis as assessments.

In finding our pedagogical feet, we felt constrained by the (lack of) value placed on QR and QM within the curricula, and also by the wider HE context where the ‘success’ of courses must be measured and accounted for. For example, MT teaches QM as a standalone course over 13 weeks and uses creative methods that allow deeper student engagement (e.g. offering a creative ‘unessay’ as assessment) and encourage wider understanding of different approaches. MT’s students rate her course very highly in terms of content and teaching, however when responding to her university’s system of teaching ‘success’ measurement, such as higher student enrolment numbers within a course, MT must formulate a deliberate narrative surrounding her ‘bespoke’ course and how numbers do not reflect its crucial importance in underpinning the University’s teaching and research strategy. Alternatively, KI has limited time for QM teaching which sits in a module that covers a wide range of research methods. Her strategy was centered on ensuring students had a solid foundation in just one method of data generation and analysis, employing tactics such as synchronous and asynchronous teaching materials, using tasks including applied practical individual and group exercises. Her students rate her teaching very highly (4.5/5) in terms of content and delivery, with most appreciating the live practical sessions that help them put their theoretical learning in practice (interviewing and coding exercises). As a result, KI negotiated to increase the time allocated to QM teaching in this module allowing more space for teaching and learning.

### Recognizing the translational applicability and value of qualitative research

Throughout our discussions we all spoke passionately about the value of QR in medicine. At the heart of the team’s motivations was the critical translational importance that QR can add such as amplifying the voices of health service end-users, elevating lived experiences within a medical model, and enhancing quality of care. That is, applying QR findings into clinical practice. Critical self-reflection was identified as one of the unique skills taught through such courses that could not only improve research in these areas but also workplace practices. In response to SW’s question about ‘what do you hope learners will gain from your course?’ MT recognized the difference between a clinical and qualitative interview and responded simply “*That they do it safely…. Honour your participants, be there for the right reasons.”* MT went on to describe her educational approach as much more than just teaching technique, passing on information or imparting new skills. MT shared that she works to instil a sense of value and self-reflection in students about the position of power that they hold as researchers, and the ethical obligations that this poses in terms of power sharing dynamics with research participants. From their institutional course ‘experience’ surveys (data not included here) MT’s students said her QM teaching helped them draw parallels between constructing rigorous QR and conducting good clinical assessment interviews. These included the critical importance of ethics in research, informed consent and protecting vulnerable patients. KI also described her tactics to encourage students to practice reflexivity and examine their own beliefs, judgments, and practices and how these may have influenced the research. She believes that reflexivity is embraced in many approaches to QR whereas those adopting quantitative methods often tend not to talk about their beliefs and biases as they are seen weaknesses:

“….you have to be reflexive in all types of research not just qualitative. If you do diabetes research and you have a diabetes diagnosis yourself, that will shape your research.” (KI).

MT described her own experiences regarding an emerging discourse between the apparent undervaluing of QR while at the same time an increasing recognition of its translational value within consumer-led medical research. She said:

“There is not a lot of status allocated to qualitative or interpretive work. But suddenly a lot of grants want lived experience, they want co-design and co-production... And I’m sitting there going ‘oh by the way guess what I can do?... guess what I have done?... and guess what I’ve been doing?’ and so there’s this big scurry to learn what this stuff is so they can put it on a grant” (MT).

Evident in MT’s account is her frustration of QR being overlooked as value work, under-resourced and her skills as a qualitative health researcher being underappreciated. Mindful of how she views her own position as a QR educator and her ever-present impressions of being undervalued by some colleagues, she acknowledges an emerging shift in how QR has been viewed in the past and is beginning to be recognized more for its translational research value in medicine.

The efforts of both MT and KI to raise the profile of QR in their own institutions resulted in some recognition by both faculties. For example, the time allocated to QM teaching in KI’s course increased from 7.5% in-person in 2018 to 30% online in 2022. She also introduced for the first time a qualitative assignment which carries equal weight (30% of the total mark) to the other two assignments (quantitative analysis and project proposal). KI a won the Dean’s award for Education because of her wider QR teaching activities. The course she teaches in won the best overall University course in 2022 demonstrating students’ satisfaction of the course and the importance of combining both qualitative and quantitative teaching and has been invited to be a qualitative supervisor/co-applicant on doctoral and clinical research grants. Furthermore, the qualitative network that she founded and has led (since 2018) has grown dramatically in 2020 indicating the increasing appetite for QR in medical research. MT is also an award-winning educator; most recently being awarded Fellow of the Advance Higher Education Academy in the UK, and receiving an Australian Award for University Teaching. The passage of time has meant even greater popularity for her as a sought-after supervisor for doctoral candidates completing qualitative studies, as a qualitative methodologist to write and review mixed methods grants, and by research teams internal to the University as well as external organizations seeking her expertise for capacity building in QR design and conduct. Recognized for her honesty in explaining QM and breaking down ‘jargon’ she is mentoring more clinicians than ever, in their qualitative pursuits and projects.

## Discussion

This article highlights the challenges faced by QR educators and shares some reflections and recommendations (Box 2) that could be helpful to those teaching QM to medical students. We also argue that some of these recommendations are relevant and could add value to teaching in general in HE. Our CAE analysis revealed the challenges that we, as educators, have faced. These can be explained in terms of contextual constraints where QR is afforded lower status than quantitative and experimental work, with the assumption that they are theoretically more closely aligned with clinical research (Guyatt et al., 2000; Bolander Laksov et al., 2017). At an institutional level, this is often the justification for the disparity in status and resources that are afforded to QR. These include the limited curriculum time allocated, as well as, support for QM teachers (Jindal et al., 2015). Typically, QR taught outside of a social science discipline is conceptualized as less rigorous or unscientific, particularly within medical sciences where there can be a lack of knowledge of the theoretical underpinnings (Mason, 2002).

Although self-reflection is not new in education (Kreber, 2010), it remains difficult to locate in-depth, critical reflections by QM educators in medical schools as to how they go about their teaching, and what influences their decisions. This could leave new educators feeling disoriented, and unsure about their own legitimacy and value in `learning environments where QR is often marginalised. Guided practice can help to counter the criticisms often pointed at QR and assist feelings of inadequacy in teaching QM, whereby problems encountered during teaching can be illuminated and reflected upon. Learnings from this current CAE constitute evidence that can be used by other educators as part of their guided practice, to inform QR and QM teaching. It can demonstrate how using sharing and reflexive relationships within teaching can work as praxis and provide QM educators with clues to help them navigate and understand what it can mean to teach QR and QM in medicine.

Fostering curiosity in students can enhance their engagement with teaching and learning, with knowledge then nurtured through genuine involvement with course content (Mogashoa, 2014). A key message from the current work was that engaging students in QR requires a focus on the added value of QM to their own practice as current or future health professionals. It may already be the case that they make use of QM, as part of their own clinical inquiries (Kajamaa et al., 2020). Experiential learning is a widely used learning process in teaching within HE whereby students ‘learn by doing’ and by reflecting on the experience, representing a learner-centric pedagogy (as opposed to teacher-centric) (Kolb & Kolb, 2012). Experiential learning within the context of teaching QM can be an effective and beneficial learning tool, such as using group-based interviews and coding assignments over more essay-based work, reading or discussion forums alone, to encourage students to work with their own capabilities and interests as well as learning through opportunities provided to them. The qualitative interviewing skills the student gains from learning about QR could improve their interactions with patients, as more emphasis is placed on patients as the experts regarding their own experiences and needs (Hunt et al., 2011).

The choice of what and how to teach QM can be governed by the time and space permitted. Shorter time frames require a more pragmatic approach, such as ensuring students have opportunities to engage in-depth with one QM. Longer teaching terms can allow greater creativity and flexibility, such as a multifaceted assignment conceptualized over many weeks. Our CAE highlighted obvious differences in our teaching approaches, in keeping with our own institutional and personal paradigms, yet identified a common student-centered approach overall. This is in keeping with typology whereby we tailored learning to the student’s position and interests in their research journey. QM teaching and learning within medicine can, ultimately, enable health professionals to inform and innovate health services and care (Tigelaar et al., 2006). But QM educators need to focus on equipping students with qualitative skills and knowledge which sustain their interest (Harackiewicz et al., 2016). For this to happen future curriculum development must consider in what ways learners benefit from exposure to a range of methods and approaches from across the qualitative-quantitative spectrum (Östlund et al., 2011).

CAE has enabled us to shed light on our practice in new ways, illuminating the hidden and tacit processes at work (Nind et al., 2015). It gave us the space to think in depth, reflect on and question our own everyday practices and to talk candidly and share our experiences with others, when there is often little time or space to do this. The collaborative discussions, reflections and sharing experiences had empowered us to keep driving change in our teaching and research practices and push for appreciation of QR in medical teaching. It also brought to the fore, the value of informal gatherings of QM educators, to share challenges and successes, in safe and supportive spaces which can guide and ‘build the field’ of QR and QM teaching (Keary et al., 2022). Reflection as an individual or community, on teaching practices, not only in teaching QM but HE more generally, is important yet an often-overlooked facet of teaching in medical education (van Lankveld et al., 2016). Existing reflection frameworks often assess teacher performance and competencies, with less attention given to working environment, or the educator’s perceptions or beliefs, identity and mission. This work has demonstrated the weight that environmental context can have on teaching, and the tensions that QM educators may contend with as they build a strong personal and teaching identity.

### Strengths and limitations

A key strength of this work includes the relationship building effect from CAE, including the longitudinal aspect of data generation, and the iterative and recursive nature of data creation by the team. Writing this article provided a welcome opportunity to pause and look back at our own teaching practices. Making use of CAE for the first time as a quartet allowed exploration of our own hidden and unspoken feelings, struggles and tacit practices. First and foremost, our discussions allowed our own voices to be raised and heard by each other, and by a wider reading audience in this manuscript. Communication was frequent (via emails and virtual meetings) and open. The team worked collaboratively and respected each other’s viewpoints and teaching and learning experiences. However, this was the first time the authors had joined together and are mindful that we did not discuss the CAE processes and group structure before commencing our group interviews and discussions.(Roy & Uekusa, 2020) Our collective reflections diary kept during the entire study period to record our thoughts, notes, and decisions enhanced the accountability and quality of our findings and provided a contemporaneous ‘mirror’ of our own preconceived notions of what QR is, and how QM should be taught. Matters of relational ethics such as confidentiality could be problematic in CAE (Lapadat, 2017). However, we as researchers and educators accept full ethical responsibility for what and how we conducted and presented the research findings including using direct and identifiable quotes from our discussions. Hernandez et al. (2017) highlighted the promise of CAE “as a critical method for fostering global collaboration that disrupts hegemonic theorizing. By listening to the voices of scholars from different parts of the world, published CAE projects could shed much-needed light on the necessity for contextualizing in theory-building”. We are grateful for the opportunity to learn more about ourselves individually and collectively as a group. We also acknowledge that this work has centered solely on the experiences of academics working in university settings, our teaching and learning conducted within western contexts, and the privileges we each hold to be able to take a self-reflective approach to our ways of teaching. Future work would benefit from extending this current dialogue to non-western qualitative teaching philosophies and experiences and inviting a wider collaborative experience with learners participating alongside the educators.

Box 2. Key recommendations for teaching QM in medicine

1. Reflect on your own teaching philosophy, and consider taking a student-centered approach to teaching QR
  To address the challenge that QR is considered of lower value than quantitative methods, focus teaching on the added value of QR to students/learners’ own practice (such as understanding patients’ experiences/beliefs)
  Practice a student-centered approach such as the communication of clear course goals and scaffolding of QR concepts to build confidence, and discipline-relevant approaches of putting QM into context for medical students, to foster a participative learning class atmosphere.
  Use evidence-based pedagogic approaches that encourage students’ active and experiential engagement with QR such as roleplaying, visual inquiry and assignments including the ‘unessay’ to encourage learners to think about research through a lens with which they are unfamiliar.
2. Consider the time, space and position of your QR teaching
  Reflect upon how the time allocated to your teaching and the positioning of your QR course in relation to other courses could impact on student’s learning.
  Be pragmatic, if necessary, especially where the time and space afforded to teach QR methods is limited and provide a considered approach to what you will focus upon. For example, choosing to engage students in depth with one approach to QR, rather than learn a little about a multitude of methods.
  Be creative and flexible to allow students to engage in learning QR through the adoption of different activities.
3. Continue to raise the profile of QR approaches and methods
  Continue to raise the importance and credibility of interpretive/qualitative works for all health professionals to access and continue to inform and innovate health and care.
  Consider working with researchers and educators from different disciplines to share rigorous approaches to QR.
4. Reflect on your experience of teaching QR methods
  Allow space and time to share and reflect on your teaching experiences. Using CAE or another self-study method could be helpful. It helped us to reflect on our practice, identify and work through the hidden and tacit processes at work, while also strengthening our advocacy of qualitative work.
  Join or establish a network or community of qualitative researchers for peer support, sharing and reflection. The qualitative network that KI leads was key to kickstarting conversations about the challenges of teaching QR methods in health and medicine and led to this international collaboration. Such networks can provide safe and supportive spaces for educators to share challenges and success stories.

## Conclusions

This work took over a year in the making and challenged the four authors to closely examine how they taught under- and post-graduate QR and QM to medical students and why they taught it in the way they did. Beginning innocuously with casual online conversations, which led to a workshop, and ultimately to longitudinal critical self-reflective dialogue through CAE—this work evidences the value of exploring shared and divergent understandings of teaching QR and QM within marginalized spaces. The authors, through interaction with each other, made in-depth discoveries about their own QM teaching and revealed an intense advocacy they each held towards pedagogical practice that sought to foster more competent and confident learners who could reflect on and apply QM to understand the behaviors and experiences of others. We hope that in reading this work, QR and quantitative educators at any career stage can utilize it as guided practice towards their own teaching and recognize their own value in student learning.

## Supplementary Information

Below is the link to the electronic supplementary material.

Supplementary file1 (DOCX 20 KB)
