# Supplementary material for: Using collaborative autoethnography to explore the teaching of qualitative research methods in medicine
Source: Adv Health Sci Educ Theory Pract. 2023 Apr 27;28(5):1467–83. doi: 10.1007/s10459-023-10224-z (PMC10136391; doi:10.1007/s10459-023-10224-z)
Supplement: Supplementary file 1 — Supplementary file1 (DOCX 20 KB) [file 10459_2023_10224_MOESM1_ESM.docx]

Supplementary file:

Topics covered across the interviews

**UNDERSTANDING THE EDUCATORS**

1) I would like to start by inviting you to provide each other, and myself, with some background about how you came to teach qualitative research methods.

*Prompts:*

- *What do you currently teach? Has this always been the case?*
- *What do you enjoy the most?*

2) How would you describe yourself as an educator?

- *Can you tell me about your pedagogical approach (e.g. active learning experiential*

*learning, student-centered, standpoint/reflexivity, collaborative learning)?*

**UNDERSTANDING THE LEARNERS**

3) I’d also like to gain a greater understanding of how you conceive of your current learners.

*Prompts:*

- *Who are you teaching? Undergraduate, postgraduate and/or continuing professional development (CPD).*
- *Have you experienced any disciplinary differences?*
- *What strategies do you use for accommodating different needs? Inclusivity?*
- *Has how you’ve conceived of your learners changed over time? Over the past year?*

**DISCIPLINARY BACKGROUND AND METHODOLOGICAL ORIENTATION**

4) Following on from reflecting on how you conceive of your learners, I’d like to turn attention to the implications of the disciplinary backgrounds and the departments/faculties in which you are located. How is qualitative research and the teaching of qualitative research methods viewed and/or positioned in your department/Faculty?

*Prompts:*

- *Can you comment on the dominant approaches in your discipline and how it shapes your practice?*
- *Have you had any alternative experiences?*
- *If so, did your approach to teaching and learning differ?*
- *If not, do you think teaching and learning qualitative methods differs between disciplines and how?*
- *Thinking about your own journeys through learning qualitative methods, can you describe any moments when you felt particularly inspired, that you have since used/adapted in your own work?*
- *How do you feel the disciplinary orientation of the educator influences the practice of teaching qualitative methods?*

**DESIGNING COURSES AND SESSIONS**

5) I’d like to turn now to the design of qualitative research methods courses and sessions and the pedagogic planning that is conducted (Mel Nind and Sarah Lewthwaite describe as ‘strategy’ in their typology of research methods teaching) What is your strategy in designing a qualitative methods programme, or a session on a particular method?

*Prompts:*

- *What do you hope learners take from the course?*
- *How do you decide:*
- *What to include?*
- *What to emphasize?*
- *What resources to source and share?*

**WHAT WORKS?**

6) Thinking about your own practice of teaching qualitative methods, ‘what works’ in your opinion?

*Prompts:*

- *Is there anything that has been particularly effective? Or challenging?*
- *What tactics (actions to put your strategy into practice) do you use?*
- *What kind of tasks do you ask/encourage learners to complete?*
- *Has your practice varied between in-person, blended and remote learning?*
- *If so, how?*
- *Has ‘what works?’ changed over the past year?*
- *How do you tell what has been effective?*
- *Tell us about your own approaches to developing an inclusive classroom*
- *What practices of reflection do you build in?*

**ASSESSMENT AND EVALUATION**

7) I’d like to turn now to thinking about assessment in more detail – and wondered how do you evaluate quality in qualitative work?

*Prompts:*

- *Has the use of formative (learning through the course) and summative assessments (what learnt at the end) changed over the last year?*

8) How do you encourage learners to evaluate quality in qualitative work?

9) Do you gather learner perspectives on your practice? If so, how – and what do you do with the feedback?

**CHANGE OVER TIME**

10) I’d like to ask you whether you feel teaching and learning has changed over time?

*Prompts:*

- *What, in your opinion has driven these changes?*
- *How is the pandemic altered how qualitative methods are taught? And learnt?*
  - *Your pedagogical approach?*
- *What occurs outside/backstage – has that shifted over pandemic.*

**TEACHING – RESEARCH INTERCONNECTENESS**

11) I’d now like to explore connections between your teaching and research. Has teaching qualitative research methods shaped your own research?

*Prompts:*

- *In what way?*
- *What have you learnt from your learners?*
- *Thinking more broadly, can teaching qualitative methodology and methods change how we do qualitative work?*

**TRAINING THE TRAINERS**

12) I’m interested in understanding how educators identify their own training needs. Do you engage in any methods-related training, CPD etc?

*Prompts*

- *Do you feel that methods training needs of staff in your institution adequately met?*
- *Building on this, what are the new emerging topics in qualitative methods that educators may need training in?*
- *Are there any specific training needs with regards to new forms of data and the changing data environment?*

**FUTURE**

13) What do you think the teaching and learning of qualitative methods might look like in the future?
